# 

**Published:** 1915-11-06

**Authors:** 


### CONTENTS.

PAGE

**Military and Medical Recruiting** ... ... ... 111

**Should Pyrexia be Treated?** ... ... ... 112

**Hospital and Institutional News** ... ... ... 113

War Effects on Voluntary Hospitals—A "Nurse Cavell Bed" at Cardiff Hospital—Babies on Exhibition—Improvements at the Middlesex—The Practices of Doctors who are at the Front—An Extraordinary Case at Bradford—A Good Example—The War and the Eastbourne Guardians—Professor Carrel's Researches—Self-Inoculation of "Gas Gangrene"—The M.A.B. and Matrimony—A Heavy Deficit at Sheffield—The Urgency Cases Hospital—The Building Schemes of Local Authorities—Charge for Visitors' Teas—The Price of Commodities in War-Time—The Doncaster Infirmary Report—The Unpaid Charitable Worker—A Hospital with a Weak Spot—The Stage and the Red Cross—Hospital Sunday in Birmingham—The Autumnal Fever Epidemic—Manchester Royal Infirmary—Higher Cost of Patients' Maintenance—Infectious Diseases and Vermin—The Strain on Small Hospitals—Exchanging Charity Votes—The Welsh Medical School—The Royal South Hants and Southampton Hospital—This Week's Drug Market.

**Eye-Strain as a Cause of Disease** ... ... 119

Factors in Successful Treatment.

**The Practitioner and the Professional Soldier** 121

Their Relations in the R.A.M.C.

PAGE

**The School Doctor** ... ... ... ... ... ... 123

The Examination of Fatigued Children.

**Personal Service in Leicester** ... ... ... 124

Some Fruits of War-Time.

**King Edward's Hospital Fund** ... ... ... 125

The Statistical Report for 1914.

**Round the World** ... ... ... ... ... 126

Fellow-Workers and the Roll of Honour.

**The Institutional Library** ... ... ... ... 127

Views on Some Social Subjects—Fatigue—Urgent Surgery—Medical Annual Synoptical Index to Remedies and Diseases—Fighting the Fly Peril—An Index of Treatment—An Index of Prognosis and End-Results of Treatment—Feeble-Minded in Ontario.

**Parliament and the Profession** ... ... ... 129

**Passing Events and Topics** ... ... ... ... 130

**Patriotism and the Nurse-Training Schools** ... 131

III.—A Group of North-Country Hospitals.

**The Editor's Letter-Box** ... ... ... ... 132

The 3rd London General Hospital.
"Frost-Bite" in the Trenches.

**Institutional Needs** ... ... ... ... ... 132

**Buildings Contemplated** ... ... ... ... 132

**The Institutional Worker** ... ... (Suppl.) 1

Work for Consumptives in Sanatoria:—Answer to September Question.—II.
Question for November ... ... (Suppl.) 2

